# The equity group: Supporting Cochrane's social responsibility of improving health equity

**DOI:** 10.1002/cesm.12012

**Published:** 2023-05-15

**Authors:** Roses Parker, Jennifer Petkovic, Jordi Pardo Pardo, Andrea Darzi, Omar Dewidar, Joanne Khabsa, Elizabeth Kristjansson, Tamara Lotfi, Olivia Magwood, Lawrence Mbuagbaw, Kevin Pottie, Alison Riddle, Ammar Saad, Eve Tomlinson, Peter Tugwell, Vivian Welch

**Affiliations:** ^1^ The Cochrane Collaboration London UK; ^2^ Bruyère Research Institute and University of Ottawa Ottawa Ontario Canada; ^3^ Department of Medicine, Faculty of Medicine University of Ottawa Ottawa Ontario Canada; ^4^ Ottawa Hospital Research Institute Ottawa Methods Centre Ottawa Ontario Canada; ^5^ Department of Health Research Methods, Evidence, and Impact McMaster University Hamilton Ontario Canada; ^6^ Department of Anesthesia, Faculty of Health Sciences McMaster University Hamilton Ontario Canada; ^7^ Michael G. DeGroote National Pain Center McMaster University Hamilton Ontario Canada; ^8^ Clinical Research Institute American University of Beirut Medical Center Beirut Lebanon; ^9^ School of Psychology, Faculty of Social Sciences University of Ottawa Ottawa Ontario Canada; ^10^ Bruyère Research Institute Ottawa Ontario Canada; ^11^ The Interdisciplinary School of Health Sciences University of Ottawa Ottawa Ontario Canada; ^12^ Department of Pediatrics McMaster University Hamilton Ontario Canada; ^13^ Biostatistics Unit, Father Sean O'Sullivan Research Centre St Joseph's Healthcare Hamilton Ontario Canada; ^14^ Centre for Development of Best Practices in Health (CDBPH) Yaoundé Central Hospital Yaoundé Cameroon; ^15^ Department of Global Health, Division of Epidemiology and Biostatistics Stellenbosch University Cape Town South Africa; ^16^ Departments of Family Medicine, Epidemiology and Biostatistics Western University London Ontario Canada; ^17^ School of Epidemiology and Public Health University of Ottawa Ottawa Ontario Canada; ^18^ Population Health Sciences, Bristol Medical School University of Bristol Bristol UK; ^19^ Clinical Epidemiology Program Ottawa Hospital Research Institute Ottawa Ontario Canada; ^20^ WHO Collaborating Centre for Knowledge Translation and Health Technology Assessment in Health Equity Bruyère Research Institute Ottawa Ontario Canada

**Keywords:** diversity and inclusion, evidence synthesis, health equity, PROGRESS‐Plus, social determinants of health, stakeholder engagement, systematic reviews

## Abstract

**Introduction:**

Health equity is a moral and ethical imperative for clinicians, researchers, policymakers, and all who use health research. Both Cochrane and the Campbell Collaboration have focused on health equity for many years.

**Methods:**

The new Equity Group will continue and expand this work by designing a program of projects aiming to (1) promote equity in the evidence base, (2) ensure equitable processes for stakeholder engagement, (3) produce high‐priority, equity‐focused evidence syntheses, (4) build capacity for equity design, analysis, and reporting, and (5) promote equity in implementation tools.

**Results:**

We will build on our current network of collaborators and create a group structure striving to recruit across the PROGRESS‐Plus characteristics.

**Conclusion:**

We invite readers to join our cause and contribute wherever they are able. Together, we can help Cochrane achieve its social responsibility of improving health equity at a planetary level.

## 1 INTRODUCTION

Health “inequalities” refer to differences in health or health outcomes [1]. Health “inequities” refer to the differences in health outcomes, which are unnecessary and avoidable, but, in addition, are considered unfair and unjust [2]. These differences in health outcomes are largely driven by socially constructed barriers to opportunities to improve health and coexist and intersect across multiple levels of society [3]. “Inequity” implies a moral and ethical dimension and health equity is a moral imperative of growing importance to all who work in health care.

The global importance of health equity was recognized by the World Health Organization Commission on the Social Determinants of Health over a decade ago [4]. The recommendations from this commission focused on the need to address health inequities both nationally and internationally. The spotlight on health inequities has grown over the past 3 years during which the coronavirus disease 2019 (COVID‐19) pandemic demonstrated how the social determinants of health can greatly impact health outcomes both within and between countries. The inequitable distribution of harms related to policies aimed at reducing the spread of COVID‐19 had the greatest impact on populations already experiencing more social disadvantages [3]. For example, those of lower socioeconomic status [5], racialized populations, and those in more precarious occupations were most affected by lockdowns [6].

Systematic reviews are recognized as one of the most efficient and reliable sources of evidence to inform health decision‐making [7]. However, to increase their relevance for evidence‐based policy decisions and decision‐makers, systematic reviews need to consider health equity [8]. Not all populations will benefit from an intervention in the same way or to the same extent [9]. Considering which populations may experience a different baseline risk or differential effectiveness of the intervention can help increase the relevance of a review for decision‐making. There are four main areas within a review in which authors can consider health equity: (1) during question formulation, (2) when deciding on methods for identifying and appraising evidence, (3) in the development of a summary of findings tables, and (4) when interpreting the findings [9].

This year will see Cochrane celebrate 30 years synthesizing health literature to produce “Trusted evidence. Informed decisions. Better health.” Archie Cochrane, whose call to have an organized report of all randomized controlled trials by speciality or subspeciality, devoted his research to improving the health of vulnerable populations. The organization he inspired, Cochrane, alongside its sister organization, Campbell, is now at a crossroads facing decisions, which could direct it to make equity a core aspect of its reorganization. To support this, Cochrane reviews applying an equity lens must move beyond establishing overall effective and ineffective interventions to instead consider “what works, for whom, and in what circumstances?” [10].

This Campbell and Cochrane Equity Methods Group has just been successfully granted status to become one of the first Cochrane Thematic Groups, herein referred to as The Equity Group. The Equity Group uses an acronym, “PROGRESS‐Plus” to define socially stratifying factors across which disadvantage can be measured: Place of Residence; Religion; Occupation; Gender or sex; Race/ethnicity/language/culture; Education; Socioeconomic Status; Social Capital; and additional characteristics such as age, sexual orientation, and disability as well as features of relationships, and time‐dependent circumstances [11]. The establishment of this thematic group demonstrates Cochrane's recognition that addressing health equity is one of the most important and impactful changes that can be made in evidence syntheses. We are committed to ensuring that all Cochrane authors consider health equity in their reviews.

## 2 OBJECTIVES OF THE EQUITY GROUP

The Equity Group aims to:

1. Promote equity in the health evidence base.
2. Ensure equitable processes for stakeholder engagement.
3. Produce high‐priority, equity‐focused evidence syntheses.
4. Build capacity for equity design, analysis, and reporting.
5. Promote equity in implementation tools.

This paper will describe how the Equity Group aims to meet these objectives and the implications this has for health equity within Cochrane, The Campbell Collaboration, and international society.

## 3 METHODS

The Equity Group will run a program of projects aiming to reduce health inequity by meeting our five objectives. To effectively achieve our objectives, we aim to collaborate widely both internally and externally with Cochrane. Not all projects are led by the Equity Group, but we aim to support any work that helps achieve our objectives. Our work plan is a call to action for readers who believe in the need to promote health equity and want to support these objectives.

The Equity Group will strengthen existing partnerships with key stakeholders in Campbell and Cochrane. We will establish new partnerships with community groups, patients, consumers, and others with lived experiences of inequity. This includes those related to PROGRESS plus characteristics or to specific conditions or lifestyles (e.g., HIV, injection drug use, commercial sex workers).

**Figure 1:**
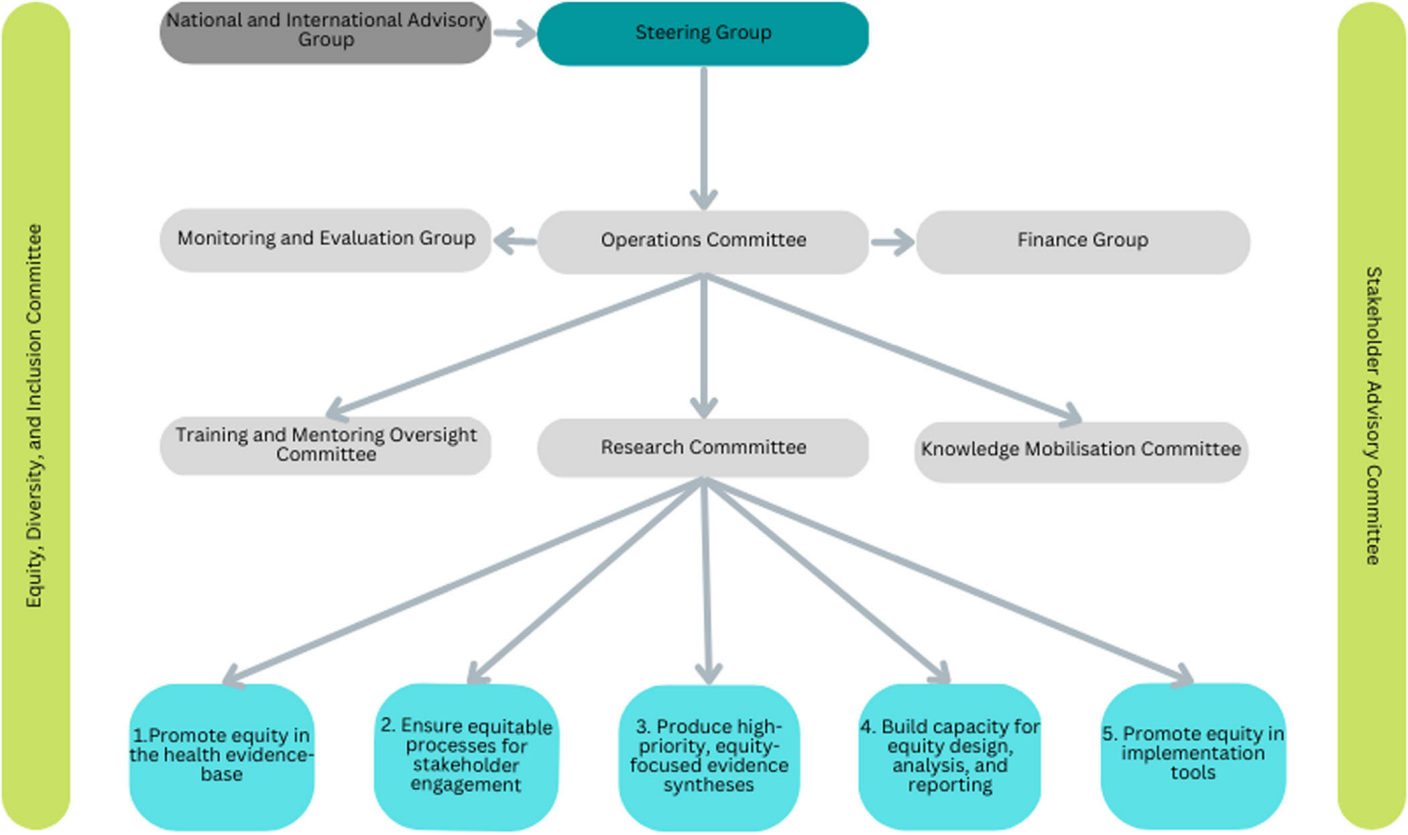
Proposed governance structure.

Our proposed governance structure is presented in Figure 1. The Operations Committee includes Convenors, Associate Convenors, Assistant Convenors, Fellows, and a Coordinator (https://methods.cochrane.org/equity/about-us). We will recruit individuals with lived experience and/or expertise in health equity topics who represent different stakeholder groups in all suitable areas of our governance structure. We will aim for diversity across PROGRESS‐Plus characteristics. Members may have expertise and/or experience across more than one topic or stakeholder group. We are in the process of expanding members of our existing governance structure from low/middle‐income countries (LMIC) including the International Board and Operations Committee. We will build on the network established by the Global Evidence Synthesis Initiative of evidence synthesis centers in LMICs. Our International Board, presented in Figure 1, will be responsible for providing input on strategic planning, conducting research on equity and disparities, and developing methods for equity in systematic reviews.

## 4 PROGRAM OF PROJECTS

The following work plan details a program of projects designed to meet our objectives (see Table 1). We have set up task groups for projects and programs of work within each objective.

**Table 1:** Projects exemplars for each objective.

Objective	Description	Area	Example projects
Promote equity in the evidence base	Providing tools, methods, and support to increase equity in observational studies, randomized trials, and systematic reviews	Equity in evidence synthesis	Racial health equity in all systematic reviews
			Equity in all Cochrane systematic reviews
		Equity in other research designs	STROBE‐Equity extension
			Operationalizing PROGRESS+ as a framework for equity‐based analyses in research
Ensure equitable processes for stakeholder engagement	Robust, inclusive stakeholder engagement for evidence synthesis and guideline development to promote equity	Stakeholder engagement	Guidance for multistakeholder engagement in evidence syntheses
Produce high‐priority, equity‐focused evidence syntheses	Priority setting for equity analysis and conducting equity‐focused evidence syntheses	Production	Conducting equity‐focused systematic reviews on priority topics
		Priority setting	Prioritizing equity‐focused systematic reviews
Build capacity for equity design, analysis, and reporting	Creating formal and informal partnerships to increase awareness. Includes training author teams and evidence synthesis units in equity analysis	Capacity building	Gender and nutrition data toolkit review
			Capacity building for evidence synthesis
			Commercial determinants of health
		Knowledge translation	E4E—Evidence for equity
Promote equity in implementation tools	Ensuring guidelines consider equity including evaluating equity analysis, developing consensus‐based guidance, updating checklists for guideline development, and engaging stakeholders in guideline development	Equity in guidelines	Equity and eCOVID Recmap
			Equity in Medical Cannabis Guidelines
			GRADE—Equity
			Equity extension of the GIN‐McMaster Guideline Development Checklist

Abbreviations: GIN, Guidelines International Network; STROBE, Strengthening the Reporting of Observational Studies in Epidemiology.

### 4.1 Promote equity in the evidence base

We will develop methods for promoting equity in observational studies, randomized trials, and systematic reviews. We will produce evidence‐based, consensus‐informed, robust extensions to widely used research tools for these methodologies. For example, we will produce a STROBE‐Equity extension [12] for observational studies following methods used previously to produce CONSORT‐Equity extension for randomized trials [13] and PRISMA‐Equity extension for systematic reviews [14].

We will disseminate these methods and use a variety of forums to ensure that systematic reviewers are trained and comfortable with using them. We aim to facilitate the application of an equity lens for every Cochrane systematic review.

In addition, we will assess the analysis and description of PROGRESS‐Plus characteristics in systematic reviews and develop guidance on approaches to integrating health equity in systematic reviews. One example of this is a project led by the Cochrane US network on centering racial health equity in systematic reviews. This project is conducting four landscape analyses on the engagement of racialized people in systematic reviews, an analysis of race and ethnicity within systematic reviews, logic models to consider systemic racism and its effects, and an analysis of terminology and definitions of racism and health equity in systematic reviews [15, 16]. This project will culminate with establishing guidance for systematic reviews and setting priorities to address evidence needs on centering racial health equity in systematic reviews.

### 4.2 Ensure equitable processes for stakeholder engagement

To be truly equitable, stakeholder engagement is important for evidence synthesis and guideline development. We aim to conduct robust, equitable, stakeholder engagement for evidence synthesis and guideline development. For example, we have received funding to develop guidance for equitable multistakeholder engagement in evidence syntheses [17]. We will develop a series of documents with recommendations for conducting, reporting, and evaluating stakeholder engagement throughout all stages of the evidence synthesis process.

### 4.3 Produce high‐priority, equity‐focused evidence syntheses

Systematic review production in health equity is an important aim of the Equity Methods Group. We are planning to conduct a robust priority‐setting process that will aim to identify the highest priority equity‐focused systematic reviews that should be produced. We will also produce equity‐focused systematic reviews for which external priority‐setting processes or stakeholders have highlighted the importance of the topic. One example of this will be a systematic review stemming from the aforementioned racial health equity project.

### 4.4 Build capacity for equity design, analysis, and reporting

The Equity Group aims to train author teams and evidence synthesis units to build capacity for equity analysis. This includes creating formal and informal partnerships globally.

Our capacity building includes a project to define an action plan to inform the development of a toolkit cocreated with stakeholders for the operationalization of the Gender‐Transformative Framework for Nutrition [18]. This work aims to expand the potential of nutrition programs to tackle gender inequalities by placing empowerment and gender equality at its center. This framework and toolkit include a scoping review of existing guidance and will support nutrition practitioners in LMIC to address the underlying determinants of malnutrition that contribute to health inequities and disempower women and girls.

In addition, our team at the London School of Hygiene and Tropical Medicine, as a part of the commercial determinants research group, are reviewing current guidance on systematic reviewing and meta‐analysis and determining at each stage where current guidance may need to shift due to considerations of the commercial determinants of health. These recommendations have been consolidated into a forthcoming paper and future work will involve a paper focused on equity and the commercial determinants of health and evidence synthesis.

Furthermore, we aim to conduct high‐quality knowledge translation to ensure our efforts in health equity promotion reach the right audience. The “E4E—Evidence for Equity” project develops collections of plain language summaries of systematic reviews and includes considerations for extrapolating the evidence for lower resource settings.

### 4.5 Promote equity in implementation tools

Guidelines are a vital part of the evidence ecosystem and must be developed equitably. To provide insight on how and to what extent guideline developers consider health equity when developing recommendations, we will evaluate equity considerations in COVID‐19 recommendations using the eCOVID‐19 RecMap, a database with a recommendation map housing over 6000 recommendations [19]. Through this evaluation, we aim to highlight well‐developed recommendations focused on disadvantaged populations on the map.

We will use consensus methods to develop equity‐focused checklists for guideline developers. For example, we plan on developing an equity extension to the widely used GIN‐McMaster Guideline Development Checklist [20] in collaboration with the Guidelines International Network (GIN) community.

We recognize the challenges that guideline developers face when considering equity in the development of guideline recommendations. Therefore, we will develop a systematic approach for collecting equity‐related evidence to inform equity considerations in guidelines. We will pilot our approach in several topics such as Medicinal Cannabis for chronic pain and pharmacological management of rheumatoid arthritis.

## 5 DISCUSSION AND CONCLUSIONS

This outline represents a comprehensive work plan of activities aimed at transforming Cochrane's output to improve health equity and reduce the likelihood of inadvertently causing inequity. The Equity Group will ensure support to increase health equity goes to people who are internal as well as external to Cochrane. We anticipate showcasing many of our activities at the upcoming Cochrane Colloquium and we invite interested readers to attend our presentations and participate in our workshops.

We are proud of the stakeholders who currently engage with us and recognize there is a need for more diversity and global representation within the group. In addition to achieving the work plan outlined above, the Equity Group aims to diversify its membership to increase the representativeness of its governance across the PROGRESS‐Plus framework. Consideration will be given to best practices for recruiting and deciding roles with a focus on ensuring that groups who face health inequities are represented. We will develop a plan to recruit leaders and members from LMICs, and a governance plan to ensure that all members have opportunities to lead projects.

The Campbell and Cochrane Equity Methods Group has successfully raised the profile of health equity as an important issue for consideration in evidence synthesis and other methodologies. The Equity Group will further advocate for applying an equity lens to systematic review production. Outputs produced by members of this group have supplied the academic community with resources to support equity increasing evidence syntheses, guidelines, and more. Furthermore, the membership of this group has grown leading to an infrastructure that can be effective in achieving the ambitious work plan set out in this manuscript.

This work plan shows how diverse the reach of equity work can be. The breadth and depth of the projects described herein illustrate not only the scale of expertise within the group but also the extent to which there is more to be done. Readers should see this as an invitation to join our cause and contribute wherever they are able (https://methods.cochrane.org/equity/; jennifer.petkovic@uottawa.ca). Addressing health equity is one of the most important and impactful things we can achieve through evidence synthesis. Together, we can help Cochrane achieve its social responsibility of improving health equity at a planetary level.

## AUTHOR CONTRIBUTIONS

**Roses Parker**: Conceptualization; data curation; formal analysis; methodology; project administration; resources; validation; visualization; writing—original draft; writing—review and editing. **Jennifer Petkovic**: Conceptualization; data curation; formal analysis; methodology; supervision; writing—original draft; writing—review and editing. **Jordi Pardo Pardo**: Conceptualization; data curation; formal analysis; methodology; supervision; writing—original draft; writing—review and editing. **Andrea Darzi**: Data curation; validation; visualization; writing—review and editing. **Omar Dewidar**: Conceptualization; validation; writing—original draft; writing—review and editing. **Joanne Khabsa**: Conceptualization; validation; writing—review and editing. **Elizabeth Kristjansson**: Conceptualization; validation; writing—review and editing. **Tamara Lotfi**: Conceptualization; validation; writing—review and editing. **Olivia Magwood**: Conceptualization; validation; writing—review and editing. **Lawrence Mbuagbaw**: Conceptualization; validation; writing—review and editing. **Kevin Pottie**: Conceptualization; validation; writing—review and editing. **Alison Riddle**: Conceptualization; validation; writing—review and editing. **Ammar Saad**: Conceptualization; validation; writing—review and editing. **Eve Tomlinson**: Conceptualization; validation; writing—review and editing. **Peter Tugwell**: Conceptualization; data curation; formal analysis; funding acquisition; methodology; resources; supervision; validation; writing—original draft; writing—review and editing. **Vivian Welch**: Conceptualization; data curation; methodology; validation; writing—original draft; writing—review and editing.

## CONFLICT OF INTEREST STATEMENT

The authors declare no conflict of interest.

## Data Availability

Data sharing is not applicable to this article as no data sets were generated or analyzed during the current study.
